# Comparative Study of the Neurotoxic Effects of Pregabalin Versus Tramadol in Rats

**DOI:** 10.1007/s12640-022-00557-9

**Published:** 2022-08-17

**Authors:** Ahmed E. Elsukary, Ahmed M. N. Z. Helaly, Amal A. El Bakary, Maha E. Moustafa, Mohammad A. El-Kattan

**Affiliations:** 1 Forensic Medicine & Clinical Toxicology Department, Mansoura Faculty of Medicine, Mansoura, Egypt; 2 Clinical Science Faculty of Medicine, Yarmouk University, Irbid, Jordan

**Keywords:** Tramadol, Pregabalin, Misuse, Neurotoxicity, Dopamine, Rats

## Abstract

In Egypt, both pregabalin and tramadol misuse increased in the last decade. Although many studies have confirmed the neurotoxic effects of tramadol, those of pregabalin are understudied. The aim of the study is to evaluate the neurotoxic effects of pregabalin compared with tramadol. Thirty male albino rats were included in this experimental study, and they were randomly allocated into three equal groups: group I (normal saline), group II (tramadol misuse), and group III (pregabalin misuse). All rats received the commenced drugs for 1 month. Open field tests were performed on the day of scarification, and after that, cortical samples were taken for immunohistochemical analysis and quantification of dopamine receptors’ gene expression. The drug misuse groups showed a significant decrease in weight gain at the end of the study. Open field testing showed the upper hand of controls regarding all of the tested parameters. Tramadol has a more negative impact on the locomotor parameters compared with pregabalin. Both drugs induced relatively low dopamine-1 receptor (D1Rs) expression to dopamine-2 receptors (D2Rs), mimicking the schizophrenia model. Both tramadol and pregabalin were associated with neurotoxic effects in male albino rats. These effects were less noticed with pregabalin. It is suggested that long-term abuse may end in psychosis.

## Introduction

Substance misuse is a common serious problem in adolescents, and it eventually leads to crucial psychosocial, medical, and legal problems (Rabie et al. 2020). Over the previous two decades, the prevalence of this problem has shown a sharp rise in Egypt due to socioeconomic instability (Yassa and Badea 2019). The prevalence of substance abuse is 0.8% in the Egyptian population aged between 15 and 64 years, according to the World Health Organization (Hussien et al. 2022).

Tramadol is a centrally acting analgesic with a weak opioid receptor agonistic action. It also inhibits serotonin and norepinephrine reuptake (Subedi et al. 2019). Since 2008, tramadol misuse has increased admissions to Egyptian addiction treatment facilities (Abolmaged et al. 2013). According to a previous Egyptian study conducted in Zagazig city, tramadol misuse was reported by 40% and 21% of temporary and permanent cleaners in the tested governmental hospitals (Abbas et al. 2013).

This problem motivated the Egyptian health authorities in 2012 to move this medication from schedule three to one on the list of highly addictive substances (Bassiony et al. 2016). Except for controlled pharmaceuticals, one can purchase any drug without a prescription in Egypt and other nations in the Middle Eastern region (Wazaify et al. 2017).

This limitation in tramadol dispensing led to a marked diversion to the misuse of other substances, including pregabalin and certain ophthalmic topical medications (e.g., cyclopentolate), which have sympathetic, anticholinergic, or antihistaminic properties (Al-Husseini et al. 2018).

Pregabalin is a centrally acting γ-aminobutyric acid (GABA) analog often prescribed for patients with neuropathic pain, epilepsy, fibromyalgia, and some anxiety disorders (Evoy et al. 2021). Off-label uses were for hypnotic-dependent insomnia, alcohol dependence, and withdrawal of benzodiazepines (Cairns et al. 2019). It binds to voltage-gated calcium channels leading to increased neuronal GABA levels (Taylor and Harris 2020). In the last years, this medication has emerged as an illicit drug with numerous reports of misuse around the world (Driot et al. 2016; Gahr et al. 2013; Grosshans et al. 2010; Halaby et al. 2015), including in Egypt (Abdelghani et al. 2020). Euphoria and withdrawal symptoms have been reported with its prolonged administration in high doses (Carrus and Schifano 2012; Chiappini and Schifano 2016; Martinotti et al. 2013; Schifano 2014).

There has been a debate regarding the potential abuse of pregabalin in the previous studies. Previous researchers noted that pregabalin was not associated with rewarding effects or a change in place preference when doses up to 30 mg/kg were administered through either the oral (Andrews et al. 2001) or intraperitoneal routes (Rutten et al. 2011).

Nonetheless, Althobaiti et al. reported that higher doses of the same drug (60 and 90 mg/kg) elicited addictive behavior in the treated rats. The previous authors attributed that effect to GLT-1 downregulation (Althobaiti et al. 2019).

From our experience at the Toxicology Unit, Mansoura University Emergency Hospital, many cases presented with an acute overdose of pregabalin who reported a history of regular chronic use of pregabalin. In April 2019, the Egyptian authorities added pregabalin to the controlled medication list (Shokry et al. 2020). Moreover, the European Union added a warning regarding its misuse to the Summary of Product Characteristics (Evoy et al. 2017).

Currently, there is an evident lack of knowledge regarding the neurotoxic properties of pregabalin. Moreover, there is a clear lack of studies comparing its harmful effect to tramadol. This was a good motive for us to conduct the present study. This work aims to track the dopaminergic receptor expression changes with pregabalin and tramadol misuse.

## Material and Methods

### Study Design

We conducted this prospective experimental research at Mansoura University following approval from the Local Ethical Committee and Institutional Review Board (IRB) of Mansoura Medical School (code number is MD/19.09.229). The current study followed the National Research Council’s Guide for the Care and Use of Laboratory Animals.

### Sample Size

The sample size was calculated using the Power Analysis Sample Size software program (PASS) version 15.0.5 for Windows, with the relative expression of dopamine-1 receptors (D1Rs) as our primary outcome. The expected difference (the effect size) was assumed to be 0.6 in the relative expression of D1Rs in the tramadol group compared to the control group, with an alpha level of 0.05 and a target power of 80%. The estimated needed sample size was 21 rats. For more accuracy, the total number increased to 30 rats (10 rats in each group).

### Animals and Experimental Protocol

We included 30 healthy adult male albino rats aged 3 to 6 months (190–210 g) in the current research. They were put in plastic cages under a standard light/dark cycle at a constant temperature of 25 °C. We used the standard laboratory diet and water for feeding. Animals were randomly divided into three groups (10 rats each). In group I (control group), each animal received orally 1 ml of normal saline per day by gavage. In group II (tramadol misuse group), each animal received a starting dose of 7.2 mg/day of tramadol (tramadol hydrochloride 225 mg in each tablet, October Pharma Co., Giza, Egypt) orally by gavage. The dose was increased by adding the starting dose every 3 days till the experiment ended (30 days) till reaching a final dose of 72 mg/day. In group III (pregabalin misuse group), each animal had received a starting dose of 10.8 mg/day of pregabalin (each capsule contains 50 mg pregabalin powder, Lyrica^®^, Pfizer, Cairo, Egypt, under license of Pfizer Inc., USA) orally by gavage. The dose was increased by adding the starting dose every 3 days till the experiment ended (30 days) till reaching a final dose of 108 mg/day. The starting dose of tramadol was equivalent to the therapeutic dose of tramadol in humans (400 mg/day) (Badawy et al. 2014), and the starting dose of pregabalin was equivalent to the therapeutic dose of pregabalin in humans (600 mg/day) (Ryvlin et al. 2008). We calculated the equivalent animal doses based on the equation of Paget and Barnes (the therapeutic dose for a rat weighing 200 g = 18/1000 × adult human therapeutic dose) (Paget and Barnes 1964). It estimates the total animal dose rather than mg/kg, supposing that the adult albino rats weigh 200 g. As all of the included animals weighed around 200 g (range, 190–209 g), we preferred to apply this formula for easy drug dosing and doubling for all animals, and that was the same way applied by the previous two relevant pieces of research (Badawy et al. 2014; Elgazzar et al. 2021).

At the end of the experiment, we measured the weight of the rats in each study group and compared it with their baseline weight. Male albino rats were subjected to the open field test at the end of the experiment to assess their locomotor activity (Huang et al. 2018).

### Sampling

Chloroform was used to anesthetize the rats before they were sacrificed. The brains were then carefully retrieved and cleaned in normal saline. The left cerebral hemispheres were fixed in 10% neutral buffered formalin and processed for light microscopic and immunohistochemical studies. The right cerebral hemispheres were used (within 24 h after sacrificing the rats) for RNA extraction.

### Reverse Transcription-Quantitative Polymerase Chain Reaction (RT-qPCR)

The expression of various messenger RNAs (mRNAs) of dopaminergic receptors of different target genes (D1, D2, D4, and D5) was performed via RT-qPCR. Total RNA was extracted using (RNeasy^®^ Mini Kit, Quiagen, Hilden, Germany, Cat. No. 74104), while the miRNA extraction was performed using (miRNeasy^®^ Mini Kit, Quiagen, Hilden, Germany, Cat. No.217004). Using a High-Capacity cDNA Reversed Transcription Kit, the extracted RNA (total RNA and miRNA) was converted to complementary DNA (cDNA) and kept at −20 °C for future research. The triple qPCR reaction mixture (total 20 l) mainly consisted of 2 μl (10 pmol/l) forward and reverse multiple gene primers, 2 μl cDNA template, and 10 μl SYBR green PCR master solution (Thermo Scientific, Lithuania). Each reaction was amplified for 40 cycles. Table 1 shows the primer sequences for the genes used in the research. The control gene’s cycle threshold (Ct) value, glyceraldehyde 3-phosphate dehydrogenase, was used to normalize the Ct values for each target gene (GAPDH). The Ct value of the control gene, glyceraldehyde 3-phosphate dehydrogenase (GAPDH), was used to normalize the Ct values for each target gene. The comparative ΔΔCT approach was used to determine the expression of several target genes. For each of the target genes, relative expression was calculated.

**Table 1:** The primer sequence of the studied genes

Dopamine D1 receptor	
Forward	5′-TCCTTCAAGAGGGAGACGAA-3′
Reverse	5′-CGCCTCCTTCAGTGCGTGGT-3′
**Dopamine D2 receptor**	
Forward	5′-TGAACCTGTGTGCCATCAGCA-3′
Reverse	5′-TTGGCTCTGAAAGCTCGACTG-3′
**Dopamine D4 receptor**	
Forward	5′-GATGTGTTGGACGCCTTTCT-3′
Reverse	5′-TCGGCATTGAAGATGGTGTA-3′
**Dopamine D5 receptor**	
Forward	5′-CCACATGATACCGAATGCAG-3′
Reverse	5′-CACAGTCAAGCTCCCAGACA-3′

### Histological and Immunohistochemical Studies

#### Light Microscopic Study

Four- to five-micrometer paraffin sections were obtained and then stained with:

- *Hematoxylin and eosin (HX&E) stains*: for the routine histological examination (Bancroft and Layton 2018).
- *Periodic acid Schiff (PAS) stain*: to assess glycogenosis and other metabolic abnormalities associated with neurodegeneration (Badawy et al. 2014; Layton and Bancroft 2018).
- *Congo red stain*: for assessing the β-pleated-sheet structure of amyloid (an indicator of immune degeneration) (Layton and Bancroft 2018; Nobakht et al. 2011).

#### Immunohistochemical Study

Paraffin sections were immunologically stained for **P53** (a marker for apoptosis and DNA integrity) (rabbit polyclonal anti-rat antibody against p53 (Abcam, ab131442; Cambridge, MA, USA)) and **Ki 67** (a marker for proliferation, neurogenesis, and regeneration) (cat number: GTX16667, Gene Tex Inc., CA, USA) (storage of the kit was done at 2–8 °C), and glial fibrillary acidic protein (GFAP) (Cat number MA5-12,023, ASTRO 6 prediluted IgG1, Thermo Fisher Scientific, Rockford, USA).

All previous antibodies were prepared and operated according to the manufacturer’s instructions.

#### Morphometric Analysis

We used ImageJ 1.47v software (National Institutes of Health, USA) to analyze the number of P53 positive cells and the percentage area of Ki 67 and GFAP immunostaining. In the analysis process, three perceptive non-overlapping fields from immunostaining sections from each rat within the three groups were assessed, taking the mean of the three readings to represent each rat.

### Statistical Analysis

The data collected were coded, processed, and analyzed with SPSS version 27 for Windows^®^. The Kolmogorov–Smirnov test was used to determine the normality of quantitative data. Parametric data were expressed as mean ± standard deviation, while the non-parametric data were expressed as median (range). To compare three groups with normally distributed quantitative variables, the one-way analysis of the variance (one-way ANOVA) was used, and the Kruskal–Wallis test was applied with non-parametric data. For all applied tests, *p*-values less than 0.05 were considered significant.

## Results

Initial body weight was statistically comparable between the three groups. Nonetheless, final body weight significantly declined in the drug misuse groups (*p* < 0.001). The percent of body weight increase was significantly higher in controls compared to the other two groups (Table 2).

**Table 2:** Analysis of studied rats’ body weight (grams) within different groups

	**Group I (control group)** **(*n* = 10)**	**Group II** **(tramadol group)** **(*n* = 10)**	**Group III** **(pregabalin group)** **(*n* = 10)**	***p*-value**
Initial body weight (gram)	201.20 ± 4.98	200.10 ± 7.25	201.60 ± 4.22	0.828
Final body weight (gram)	245.30 ± 6.85	219.10 ± 6.31 #	238 ± 5.52 # $	< 0.001
Percent of change in the body weight (%)	22.02 ± 5.37	9.55 ± 2.51 #	18.11 ± 4.01 $	< 0.001

#Statistically significant to group I (control group)

$Statistically significant to group II (tramadol group)

Open field testing showed the superiority of the control group regarding all of the tested parameters. It was also evident that tramadol negatively affected the open field-testing variables more than the pregabalin effect.

The speed, the total distance traveled, and the number of line crossings showed a statistically significant decrease in the tramadol and pregabalin groups compared to the control group. Also, these parameters showed a statistically significant decrease in the tramadol group compared to the pregabalin group.

On the other hand, the number of immobile episodes and the time spent in the peripheral zone showed a statistically significant increase in the tramadol and pregabalin groups compared to the control group. Also, these parameters showed a statistically significant increase in the tramadol group compared to the pregabalin group (Table 3).

**Table 3:** Open field test results of the studied rats within different groups

	**Group I (control group)** **(*n* = 10)**	**Group II (tramadol group)** **(*n* = 10)**	**Group III (pregabalin group)** **(*n* = 10)**	***p*-value**
Speed (cm/s)	2.21 ± 0.19	0.81 ± 0.11 #	1.50 ± 0.12 # $	< 0.001
Total distance traveled (m)	6.62 ± 0.58	2.41 ± 0.33 #	4.50 ± 0.37 # $	< 0.001
Number of line crossing	54.50 ± 7.06	20.70 ± 2.75 #	35.30 ± 5.83 # $	< 0.001
Number of immobile episodes	6 (4–9)	16 (12–17) #	10 (7–13) # $	< 0.001
Time in the peripheral zone (seconds)	240.65 ± 6.40	286.33 ± 8.05 #	272.84 ± 4.23 # $	< 0.001

#Statistically significant to group I (control group)

$Statistically significant to group II (tramadol group)

The tested four genes showed statistically different expressions among the three groups in the dopaminergic gene expression. Both dopamine-1 receptor (D1Rs) and dopamine-5 receptor (D5Rs) expressions showed a significant decline in groups II and III compared to controls. However, the expression decreased in association with tramadol (group II).

On the other hand, dopamine-2 receptors (D2Rs) and dopamine-4 receptors (D4Rs) showed a significant increase in both drug misuse groups compared to controls. This increase was more noticeable in group II compared to group III.

Both tested pharmaceuticals significantly impacted the dopaminergic gene expression, either by increase or by decrease, according to the receptor type. In all circumstances, the change was much greater in the tramadol group compared to the pregabalin one (Table 4).

**Table 4:** Analysis of the relative expression of dopaminergic receptors gene as measured by real-time PCR of the studied rats within different groups

	**Group I (control group)** **(*n* = 10)**	**Group II (tramadol group)** **(*n* = 10)**	**Group III (pregabalin group)** **(*n* = 10)**	***p*-value**
D1Rs	1 (0.99–1.01)	0.38 (0.22–0.56) #	0.52 (0.42–0.84) # $	< 0.001
D2Rs	1.01 (0.98–1.02)	3.03 (1.87–3.93) #	1.84 (1.06–2.37) # $	< 0.001
D4Rs	1.01 (0.99–1.02)	3.45 (1.06–5.74) #	1.5 (1.02–3.3) #	< 0.001
D5Rs	1 (0.99–1.02)	0.64 (0.44–0.85) #	0.70 (0.44–0.89) # $	< 0.001

#Statistically significant to group I (control group)

$Statistically significant to group II (tramadol group)

*D1Rs* dopamine-1 receptors, *D2Rs* dopamine-2 receptors, *D4Rs* dopamine-3 receptors, *D5Rs* dopamine-5 receptors

**Fig. 1:**
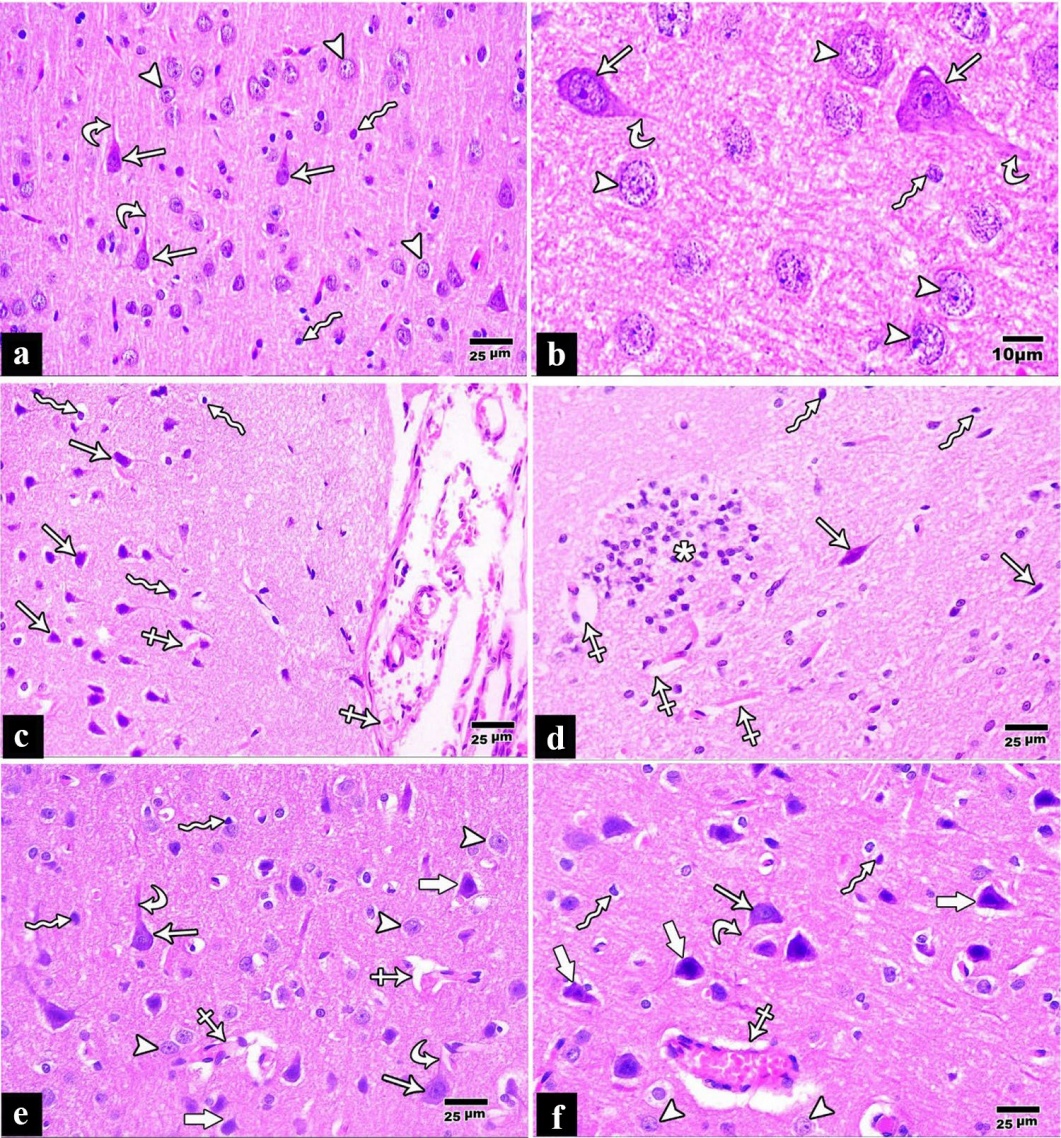
**a** A photomicrograph of a paraffin section in a control rat cerebral cortex demonstrating pyramidal cells “Arrows” with rounded open face nuclei and long apical dendrites “Curved arrow”. Granular cells “Arrow heads” with round open face nuclei are demonstrated. Neuroglial cells “Wavy arrows” are also demonstrated (H&E × 400). **b** H&E × 1000. **c**, **d** A photomicrograph of a paraffin section in a rat cerebral cortex of tramadol group showing many apoptotic cells surrounded with empty spaces “Wavy arrows,” shrunken pyramidal cells surrounded by empty spaces “Arrows,” dilated and congested blood vessels are also seen “Crossed arrows,” and inflammatory cell infiltration (asterisks) is seen in the surrounding neuropil (H&E × 400). **e**, **f** A photomicrograph of a paraffin section in a rat cerebral cortex of pregabalin group showing shrunken pyramidal cells surrounded by empty spaces “Thick Arrows.” However, apparently, normal pyramidal cells “Arrows” with their apical dendrites “Curved arrows” are also seen. Granular cells “Arrow heads” and neuroglial cell “Wavy arrows” are also seen. Congested blood vessels are also seen “Crossed arrows” (H&E × 400)

The cerebral cortex H&E-stained sections of the control group demonstrated that each pyramidal cell had a basophilic cytoplasm, long apical dendrite, and a large vesicular nucleus. Still, the granular cells had round cell bodies and a large round open face nucleus. Darkly stained nuclei of neuroglial cells in the intercellular layer were also visible (neuropil). The surrounding areas contained glial cells, nerve fibers, and blood vessels (Fig. 1a, b).

In the tramadol group, the pyramidal cells are shrunken with darkly stained nuclei and surrounded by empty spaces. The granular cells had reduced in size and were surrounded by vacant areas. Apoptotic cells were also seen, which were shrunken and had small, darkly stained nuclei and little acidophilic cytoplasm (Fig. 1c, d). Contrarily, the neuropil showed inflammatory cellular infiltrate (Fig. 1d). There were perineuronal empty spaces and vascular congestion (Fig. 1c, d).

While in the pregabalin group, some pyramidal and granular cells appeared normal, while others were shrunken with dark nuclei and surrounded by empty spaces. There were perineuronal empty spaces and vascular congestion (Fig. 1e, f).

**Fig. 2:**
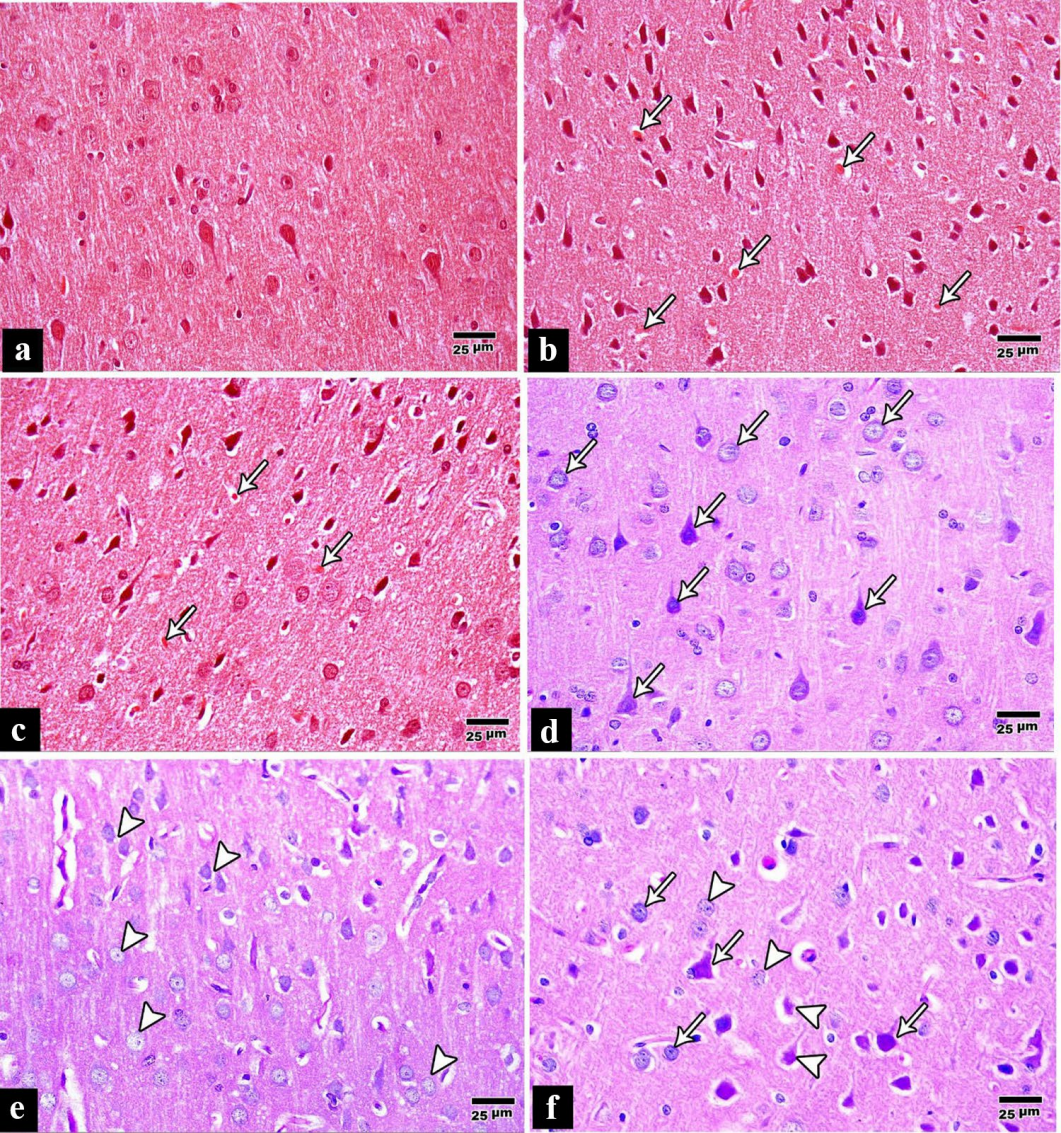
**a** A photomicrograph of a paraffin section in a control rat cerebral cortex with no amyloid deposits detected (Congo red × 400). **b** A photomicrograph of a paraffin section in a rat cerebral cortex of tramadol group showing numerous amyloid deposits “Arrows” (Congo red × 400). **c** A photomicrograph of a paraffin section in a rat cerebral cortex of pregabalin group showing few amyloid deposits “Arrows” (Congo red × 400). **d** A photomicrograph of a paraffin section in a control rat cerebral cortex demonstrating strong PAS reactions in the pyramidal and granular cells “Arrows” (PAS × 400). **e** A photomicrograph of a paraffin section in a rat cerebral cortex of tramadol group demonstrating a weak PAS reaction in the degenerated pyramidal and granular cells “Arrow heads” (PAS × 400). **f** A photomicrograph of a paraffin section in a rat cerebral cortex of pregabalin group demonstrating a weak PAS reaction in the pyramidal and granular cells, especially the degenerated ones “Arrow heads” and moderate PAS reaction in other pyramidal and granular cells “Arrows” (PAS × 400)

**Fig. 3:**
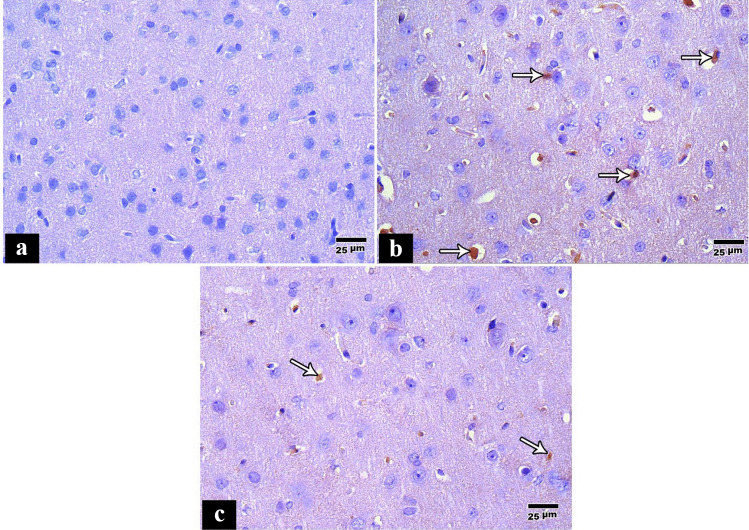
**a** A photomicrograph of a paraffin section in a control rat cerebral cortex showing a negative P53 reaction (anti P53 immunostain × 400). **b** A photomicrograph of a paraffin section in a rat cerebral cortex of tramadol group showing numerous P53 positive cells “Arrows” (anti P53 immunostain × 400). **c** A photomicrograph of a paraffin section in a rat cerebral cortex of pregabalin group showing few P53 positive cells “Arrows” (anti P53 immunostain × 400)

Examination of Congo red-stained sections of the control group revealed dull staining of neurons and neuropil without amyloid deposits (Fig. 2a). In the tramadol group, it revealed more strongly + ve masses called amyloid plaques (Fig. 2b). In contrast, in the pregabalin group, it revealed a few + ve masses called amyloid plaques (Fig. 3c).

Examination of PAS-stained sections of the control group revealed normal PAS reaction (purplish-red), which appeared strong in the pyramidal cells and the granular cells (Fig. 2d), in the tramadol group, revealed a very weak PAS reaction in neuronal cells (Fig. 2e) while in the pregabalin misuse group revealed weak PAS reaction in the pyramidal and granular cells specially degenerated ones and moderate reaction in the normal ones (Fig. 2f).

Immunohistochemical staining of the cerebral cortex of the control group showed negative immunohistochemical staining for p53. Thus, neurocytes appeared blue (Fig. 3a). In contrast, in the tramadol group, the cerebral cortex showed more p53 positive cells in the rat cerebral cortex (Fig. 3b), and there are few positive p53 cells in pregabalin group compared to tramadol group (Fig. 3c).

**Fig. 4:**
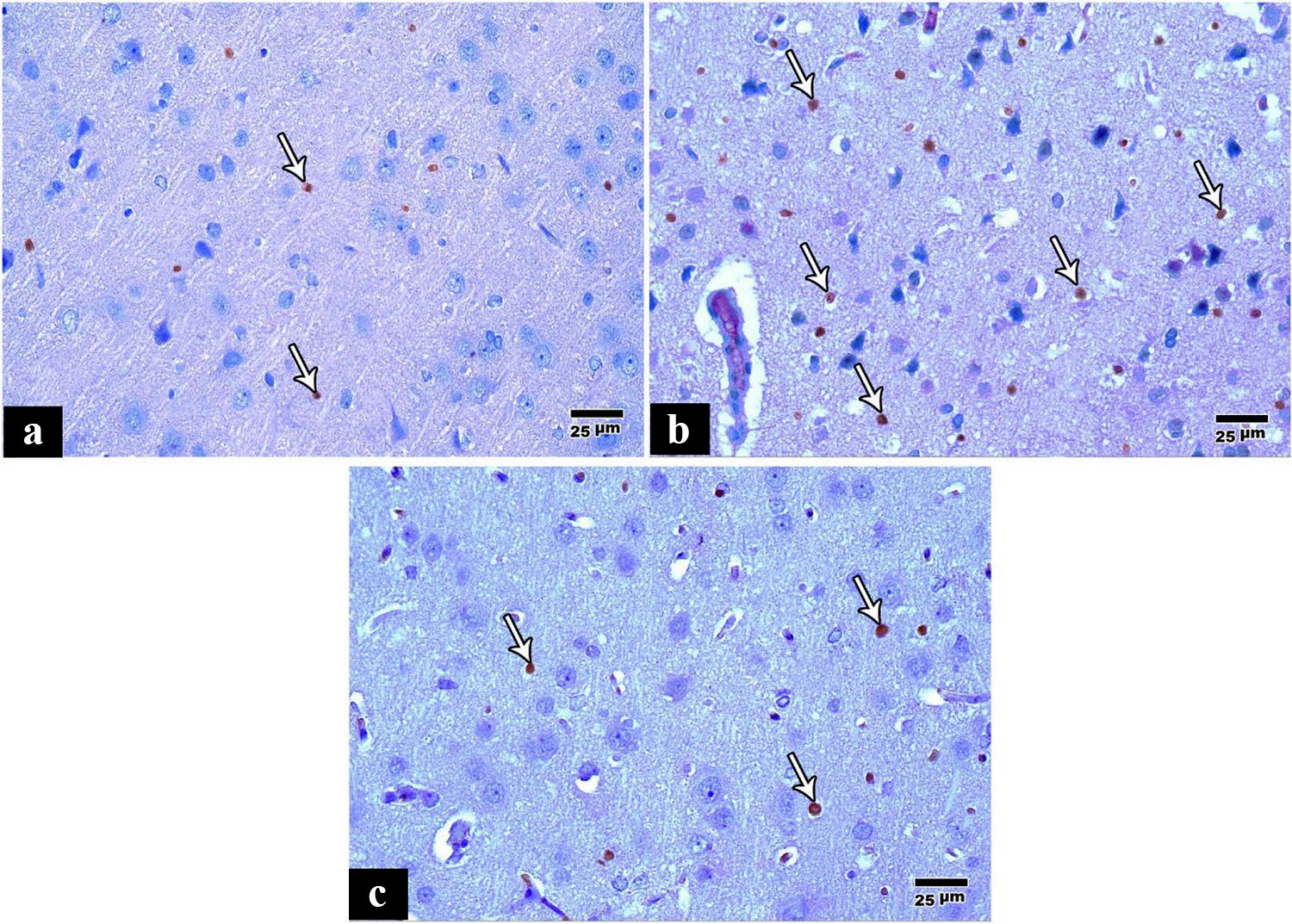
**a** A photomicrograph of a paraffin section in a control rat cerebral cortex showing some positive cells (anti Ki 67 immunostain × 400). **b** A photomicrograph of a paraffin section in a rat cerebral cortex of tramadol group showing more positive cells and increased staining intensity with mitotic figures (anti Ki 67 immunostain × 400). **c** A photomicrograph of a paraffin section in a rat cerebral cortex of pregabalin group showing more positive cells as compared to the control group (anti Ki 67 immunostain × 400)

Immunohistochemical staining of the cerebral cortex of the control group showed that some cells in the cerebral cortex expressed positive nuclear immunostaining for Ki 67 (Fig. 4a), while in the tramadol group, the cerebral cortex showed more Ki 67 positive cells and intense staining with mitotic figures (Fig. 4b). In the pregabalin group, there were more Ki 67 positive cells compared to the control group. Still, it was less than the tramadol group (Fig. 4c).

**Fig. 5:**
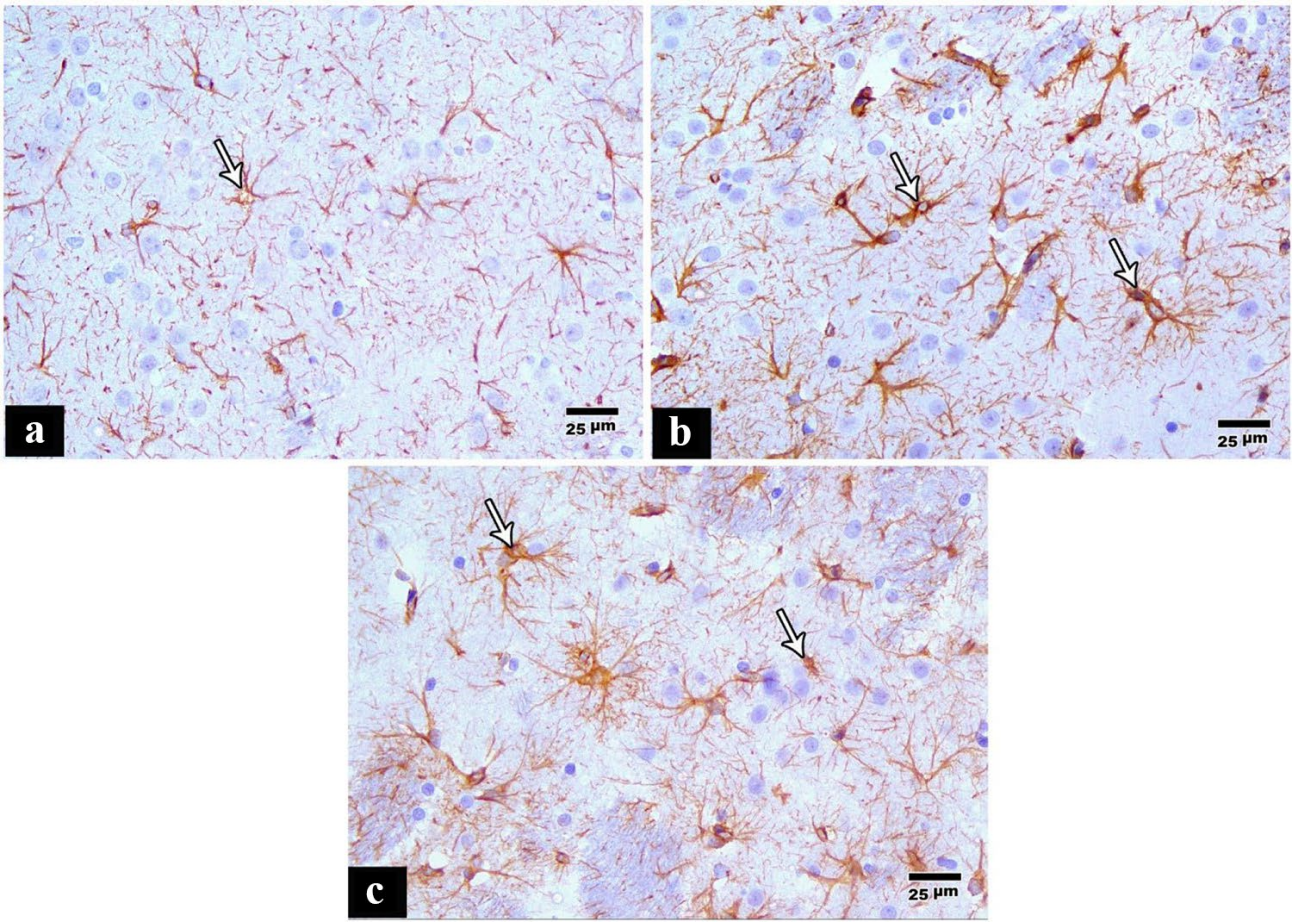
**a** A photomicrograph of a paraffin section in a control rat cerebral cortex showing some positive GFAP immunostaining in the star-shaped glial cells and their processes (anti GFAP immunostain × 400). **b** A photomicrograph of a paraffin section in a rat cerebral cortex of the tramadol group showing more positive GFAP immunostaining in star-shaped glial cells with increased branches of their cytoplasmic processes (anti GFAP immunostain × 400). **c** A photomicrograph of a paraffin section in a rat cerebral cortex of the pregabalin group showing more positive GFAP immunostaining in star-shaped glial cells as compared to the control group (anti GFAP immunostain × 400)

Immunohistochemical staining of the cerebral cortex of the control group showed positive GFAP immunostaining in star-shaped glial cells and their processes (Fig. 5a). Immunohistochemical staining of the cerebral cortex of the tramadol group showed an increase in the percentage of positive GFAP immunostaining in star-shaped glial cells with increased branches of their cytoplasmic processes (Fig. 5b). In the pregabalin group, the percentage of reaction was more compared to the control group, but less than the percentage of reaction was less than the tramadol group (Fig. 5c).

The number of positive P53 apoptotic cells, the percentage area of positive GFAP immunoreaction, and the percentage area of positive Ki 67 immunoreactions showed a statistically significant increase in the tramadol and pregabalin groups compared to the control group. Also, these parameters showed a statistically significant increase in the tramadol group as compared to the pregabalin group. The tramadol group showed higher positivity for all the tested stains, followed by the pregabalin one, then controls (Table 5).

**Table 5:** Analysis of the number of positive P53 apoptotic cells of the studied rats within different groups

	**Group I (control group)** **(*n* = 10)**	**Group II (tramadol group)** **(*n* = 10)**	**Group III (pregabalin group)** **(*n* = 10)**	***p*-value**
Number of positive P53 apoptotic cells	0	15.50 ± 2.12 #	8 ± 1.56 # $	< 0.001
Percentage area of positive GFAP immunoreaction	8.02 ± 1	38.13 ± 6.17 #	19.67 ± 3.11 # $	< 0.001
Percentage area of positive Ki 67 immunoreaction	0.34 ± 0.08	1.47 ± 0.12 #	0.60 ± 0.12 # $	< 0.001

#Statistically significant to group I (control group)

$Statistically significant to group II (tramadol group)

## Discussion

The current research was conducted to study the neurotoxic effects of pregabalin misuse and to compare it with tramadol in rats. To the best of our knowledge, this is the first study comparing the previous two drugs regarding their neurotoxicity. This unique comparison poses an advantageous point in favor of our study.

First, one could notice no significant differences between the three study groups regarding their pre-procedural weight, indicating the proper randomization. This should also negate any bias skewing our findings in favor of one group rather than the others.

Regarding the starting dose commenced in both groups, we preferred to estimate the total animal dose rather than the mg/kg dose. Most clinicians and patients prefer a fixed-dose regimen over a weight-based one, as it is more convenient and easier to prescribe (Pan et al. 2016). That is the way in the two tested drugs in our investigation, as most doses reported in the literature are adjusted according to day rather than body weight. Also, most papers reporting pregabalin or tramadol administration, whether for therapeutic or addictive purposes, express their doses via the same concept (Gajraj 2005; Lancia et al. 2019; Lanier et al. 2010; Roussin et al. 2015).

Herein, we tried to commence the rats on an escalating dose of pregabalin till reaching doses equivalent to the very high maximum daily doses reported in humans with pregabalin misuse (≤ 7500 mg/day; ≤ 3–20 × over the normal therapeutic range) as reported by Lancia and his associates. One should note that the maximum dose reached was 6000 mg per day instead of 7500 mg, as we preferred to commence an additional therapeutic dose every 3 days for easy dose calculation (Lancia et al. 2019). Regarding tramadol, as previously reported, doses up to 6 g per day were used in patients with substance abuse (Roussin et al. 2015).

These neurotoxic effects were induced mostly by the increased doses of the administered drugs. That is the main problem in individuals with substance misuse, who usually have to increase their daily dose to reach the same effect because of drug tolerance.

In the tramadol group, we also decided to increase the drug dose in the same manner as the pregabalin misuse group for an easier drug-increase protocol, and the maximum dose commenced was still in the previously reported range when the equivalent human dose was estimated.

Our findings showed a significant decrease in the final body weight with tramadol misuse compared to control. That confirms a previous study by Shuey and his coworkers (2008). The decrease in weight gain in rats receiving tramadol may be attributed to its gastrointestinal side effects, including loss of appetite (Jolobe 2015).

Generally, when looking at the present findings, a significant decrease in the locomotor activity in the tramadol misuse rats compared to controls can be noticed. That was evident in all subtests of the open field test. By the current findings, repeated administration of tramadol (50 mg/kg, oral, for 21 days) had a considerable effect on the locomotor activity in rats in the form of lower spontaneous locomotor activity (Aghajanpour et al. 2020). A prior study found that tramadol 5 mg/kg did not elicit significant locomotor changes when given once compared to the control group. Still, the higher dosages (10 and 20 mg/kg) resulted in a dose-dependent decline in locomotor activity (Szkutnik-Fiedler et al. 2012). This may be explained by the fact that tramadol caused a series of events responsible for neurodegeneration, including inflammation and microglial proliferation (mostly in the prefrontal cortex) (Aktas et al. 2007). Some studies using rodent models have shown that tramadol administration can impair memory functions by activating μ-opioid receptors (Baghishani et al. 2018; Hosseini-Sharifabad et al. 2016).

Our findings showed a significant increase in the time spent in the peripheral zone in the tramadol misuse group. The open field test assesses anxiety and locomotor activity (Seibenhener and Wooten 2015). Multiple reports have documented the association between drug misuse and anxiety (Alves et al. 2020; Lai et al. 2015). Anxiety, along with the decreased locomotor activity in the drug misuse groups, could explain the decreased tendency of our animals to spend more time in the central zone.

Regarding dopaminergic receptors, the current findings showed increased expression of D2Rs and D4Rs associated with tramadol misuse. Conversely, it led to a significant decline in D1Rs and D5Rs expressions compared to controls. According to current research, drug-induced neurotoxicity is caused by the activation of many neurotransmitter systems, including dopamine, which work together to cause brain damage (Cadet et al. 2014).

Dopamine is a neurotransmitter and hormone that is classified as a monoamine catecholamine. It binds to the dopamine receptor and has a variety of actions depending on the receptor type. Dopamine receptors are important in everyday living tasks. Movement, emotions, and the brain’s reward system are all affected by this hormone and its receptors (Schultz 2015). The known five dopamine receptors can be enrolled under two main categories: D1Rs and D5Rs group together; and D2Rs and D3Rs, in addition to D4Rs, are together in a separate subgrouping (Yu et al. 2019).

The present findings corroborated those of Faron-Górecka et al. They that repeated tramadol administration (20 mg/kg i.p. for 21 days) resulted in a considerable upregulation of D2Rs and D3Rs receptors in the rat nucleus accumbens (Faron-Górecka et al. 2004).

The D5Rs appear to perform some of the same functions as D1Rs. Both D1Rs and D5Rs play important roles in conferring the qualities of reward and novelty to information processed by the hippocampus. They modulate hippocampal long-term potentiation and memory in the brain (Hansen and Manahan-Vaughan 2014). Selective D5R agonists have recently been shown to protect neurons from apoptosis and improve cognitive function (Shen et al. 2016). Thus, its decline associated with tramadol misuse is a clear and strong mark of increased apoptosis and impaired neurological function.

In the same context, the number of apoptotic cells stained positive for P53 increased significantly in the current study. Oxidative stress, genotoxic chemicals, and other conditions cause p53 to accumulate in the nucleus and bind to certain DNA sequences, causing activation of transcription of many apoptosis-related genes (Almog and Rotter 1997). Chronic opioid treatment in rats has been linked to a significant increase in the pro-apoptotic receptor and intracellular pro-apoptotic components, as previously described (Sharifipour et al. 2014).

In line with the current findings, a prior investigation found that a greater tramadol dose significantly increased p53 gene expression compared to control rats (Mohamed and Mahmoud 2019). Additionally, Aghajanpour and his colleagues reported that the caspase-3 level, an apoptotic marker, showed a 3.5-fold increase in the rats treated with tramadol than in controls (Aghajanpour et al. 2020).

At the same time, tramadol intake in the current study led to a significant rise in GFAP immunoreaction, indicating an increased number of glial cells in the central nervous system (CNS). That agrees with Aghajanpour et al.’s (2020) results. The proposed mechanism is that microglial cells are the main mediators of the neuroinflammatory process in the CNS (Liu et al. 2015). It is responsible for inflammatory cytokine release leading to neurodegeneration (Hoogland et al. 2015). On their migration and activation, an inflammatory response is initiated within the prefrontal cortex (Dheen et al. 2007).

In the current investigation, tramadol intake was associated with a significant increase in Ki 67 immunoreaction. That does not mean an underlying proliferation of functioning neurons, as neuronal cells do not regenerate. Thus, the increased proliferation marker applied in the current study reflects the astrogliosis resulting from brain injury.

Regarding pregabalin and its effects, the current findings showed a decreased final body weight in the pregabalin misuse rats. Following the current findings, Elgazzar et al. noted a statistically significant decrease in the animal weight in the pregabalin-dependent group (after 3 months) compared to controls (Elgazzar et al. 2021). The authors attributed the weight loss to the striking lack of interest in their food intake. Also, Shokry et al. confirmed the previous findings (Shokry et al. 2020).

Regarding the open field tested parameters, we noticed a significant decline in all locomotor parameters in the pregabalin group compared to the controls. One could attribute this decline to the sedative effect of pregabalin. Our results could be explained by the reported dose-dependent sedative impact of pregabalin administration in clinical investigations (El-Hussiny et al. 2018). The inhibitory impact of pregabalin on the release of excitatory neurotransmitters in the brain has been linked to its sedative effect (Calandre et al. 2016). According to previous neuropharmacological research, pregabalin activates presynaptic voltage-gated calcium channels, with a subsequent decrease of calcium influx into nerve terminals in rats and humans (Fink et al. 2002, 2000).

The effect of pregabalin on dopaminergic receptors is understudied in the literature. We noticed that pregabalin induced the same dopaminergic receptor changes as tramadol but with a weaker effect, indicating that pregabalin still has a neurotoxic effect. Still, it is less severe than tramadol.

The authors noted a significant increase in positive P53 apoptotic cells in the pregabalin group compared to controls. Previous research reported a significant decline in B-cell lymphoma 2 (BCL2) in the pregabalin-dependent group compared to controls. Additionally, they noticed a significant increase in the inducible nitric oxide synthase, a marker of oxidative stress (Elgazzar et al. 2021). The presence of reactive oxygen species is considered an early marker of cellular apoptosis, as reported by earlier studies (Su et al. 2019).

In the same context, in the study conducted by Sayin and Simsek, administration of a supratherapeutic dose of pregabalin was associated with an increased expression of c-Jun N-terminal kinase (JNK), which is a potent initiator of both extrinsic and mitochondrial intrinsic apoptotic pathways (Sayin and Simsek 2018). Additionally, according to Salem et al. high dosages of pregabalin resulted in a considerable rise in apoptotic markers, as seen by increased caspase 3 and a decrease in BCL2 (Salem et al. 2021).

Contrarily, Song et al. treated rats with an intraperitoneal injection of pregabalin (30 mg/kg) or an equal amount of normal saline at the onset of reperfusion. The pregabalin-treated group showed fewer apoptotic cells (by 63%) compared to the control, which was evident in BCL2 upregulation and caspase-3 downregulation (Song et al. 2017). Hindmarch et al. discovered that pregabalin (30 mg/kg intraperitoneally at 30 min, 12, 24, and 48 h) lowered caspase-3 and phosphorylated p38 mitogen-activated protein kinase (MAPK) expression following spinal cord injury in rats, but BCL2 exhibited no significant difference between control and treated groups (Hindmarch et al. 2005). The disagreement between the current findings and those researchers is most likely due to differences in pregabalin dosage, duration, and route of administration.

The current findings showed a significant increase in the area stained positive for GFAP in the pregabalin group compared to controls, indicating more gliosis. As mentioned before, gliosis is a reactive response to brain injury which is indicative of pregabalin’s harmful effect (Livne-Bar et al. 2016). Using a different marker for gliosis (nestin), Elgazzar and her associates noted a significant increase in gliosis in pregabalin-dependent animals (Elgazzar et al. 2021). Of course, the increased Ki 67 expression was secondary to the astrogliosis.

When looking at the tramadol and pregabalin effects, it is evident that tramadol was associated with a more neurotoxic effect than pregabalin. However, the difference between the pregabalin group and controls was also significant regarding most of the study variables. Therefore, the neurotoxic effect of pregabalin should be considered, and its prescription should be limited only to indicated cases and under strict supervision.

Notably, low D1Rs and relatively high D2Rs expression are well-known markers of schizophrenia (Avery and Krichmar 2015). Another cornerstone pathology indicator of psychosis is neuroinflammation, which is well marked in the tramadol group compared to the pregabalin group (Comer et al. 2020). Despite the different mechanisms of action, both drugs induced common psychosis-related mechanisms. Moreover, studies showed that human data expressed evident psychosis in patients suffering from tramadol withdrawal and other addictive drugs like opioids (Lozano-López et al. 2021; Rajabizadeh et al. 2009). These findings may explore a common model for psychosis.

The limited sample size included is one of its major limitations. Therefore, this subject should be extensively evaluated in larger-sample studies.

## Conclusions

Based on the current findings, both tramadol and pregabalin were associated with neurotoxic effects in male albino rats. These effects were less noticed with pregabalin. It is suggested that long-term abuse may end in psychosis. Further research is required, in animals and humans, to explain the neurotoxic effects of pregabalin abuse in the prefrontal cortex.
